# Isolation of Flavonoids from *Deguelia duckeana* and Their Effect on Cellular Viability, AMPK, eEF2, eIF2 and eIF4E

**DOI:** 10.3390/molecules21020192

**Published:** 2016-02-06

**Authors:** Lorena M. C. Cursino, Nerilson M. Lima, Renato Murillo, Cecilia V. Nunez, Irmgard Merfort, Matjaz Humar

**Affiliations:** 1 Bioprospecting and Biotechnology Lab, National Institute of Amazonian Research, Manaus 69067-375, Brazil; lorena.cursino@gmail.com (L.M.C.C.); nerilsonmarques@gmail.com (N.M.L.); cecilia@inpa.gov.br (C.V.N.); 2 Institute of Pharmaceutical Science, Department of Pharmaceutical Biology and Biotechnology, University of Freiburg, Freiburg 79104, Germany; 3 Escuela de Química and CIPRONA, Universidad de Costa Rica, 2060 San José, Costa Rica; renato_murillo@hotmail.com

**Keywords:** *Deguelia duckeana*, Fabaceae, flavonoids, eukaryotic elongation and initiation factor 2, AMPK

## Abstract

Preparations of *Deguelia duckeana*, known in Brazil as timbó, are used by indigenous people to kill fish. Reinvestigation of its extracts resulted in the isolation and identification of 11 known flavonoids identified as 3,5,4’-trimethoxy-4-prenylstilbene (**1**), 4-methoxyderricidine (**2**), lonchocarpine (**3**), 4-hydroxylonchocarpine (**4**), 4-methoxylonchocarpine (**5**), 5-hydroxy-4’,7-dimethoxy-6-prenylflavanone (**6**), 4’-hydroxyisolonchocarpine (**7**), 4’-methoxyisolonchocarpine (**8**), 3’,4’,7-trimethoxyflavone (**9**), 3’,4’-methylenedioxy-7-methoxyflavone (**10**), and 2,2-dimethyl-chromone-5,4’-hydroxy-5’-methoxyflavone (**11**). Except for **1**, **3**, and **4** all of these flavonoids have been described for the first time in *D. duckeana* and the flavanone **6** for the first time in nature. Compounds **2**, **3**, **4**, **7**, **9**, and **10** were studied for their potential to induce cell death in neuronal SK-N-SH cells. Only the chalcone **4** and the flavanone **7** significantly induced lactate dehydrogenase (LDH) release, which was accompanied by activation of caspase-3 and impairment of energy homeostasis in the MTT assay and may explain the killing effect on fish. Interestingly, the flavone **10** reduced cell metabolism in the MTT assay without inducing cytotoxicity in the LDH assay. Furthermore, the flavonoids **2**, **3**, **4**, **7**, and **10** induced phosphorylation of the AMP-activated protein kinase (AMPK) and the eukaryotic elongation factor 2 (eEF2). The initiation factor eIF4E was dephosphorylated in the presence of these compounds. The initiation factor eIF2alpha was not affected. Further studies are needed to elucidate the importance of the observed effects on protein synthesis and potential therapeutic perspectives.

## 1. Introduction

Flavonoids possess a broad variety of different biological activities, among which their antioxidative properties are of special interest. Reactive oxygen radicals are harmful to biomembranes, but can additionally play a role as mediators in different signaling pathways [1,2]. Depending on their structural features, flavonoids such as prenylated flavones or chalcones, can also be involved in cytotoxic processes [3,4].

The genus *Deguelia* which belongs to the Fabaceae family can be found in tropical South America and shows a predominance of prenylated flavonoids and stilbenes [5]. Prenylated stilbenes as well as chalcones and isoflavonoids have been isolated from *Derris rariflora* (syn. *Deguelia nitidula*) [6]. Similar compounds have also been found in the roots of *Deguelia* *hatschbachii* [7]. Stilbenes with a slight effect on seed germination and plant growth have been described from the leaves of *Deguelia refuescens* var. *urucu* [8], whereas prenylated isoflavonoids with a slight antibacterial and antifungal activity have been reported from *Deguelia longeracemosa* [9]. In leaves of *Deguelia utilis* prenylated chalcones and stilbenes, which have cytoprotective properties in a neuronal cell line, have been found [10].

Preparations of *Deguelia duckeana* (Fabaceae) are known in Brazil as timbó and used by indigenous people for killing fish. However, the compounds responsible for these cytotoxic effects as well as their mode(s) of action are still unknown. Initial phytochemical investigations revealed three chalcones and a stilbene derivative as constituents, but no studies on their cytotoxic activities have been performed [11]. This prompted us to reinvestigate *D. duckeana* and to perform the first studies on which way the isolated flavonoids may affect cell life using the neuronal cell line SK-N-SH.

## 2. Results and Discussion

### 2.1. Isolation and Identification of Flavonoids from D. duckeana Including a New Flavanone

**Figure 1:**
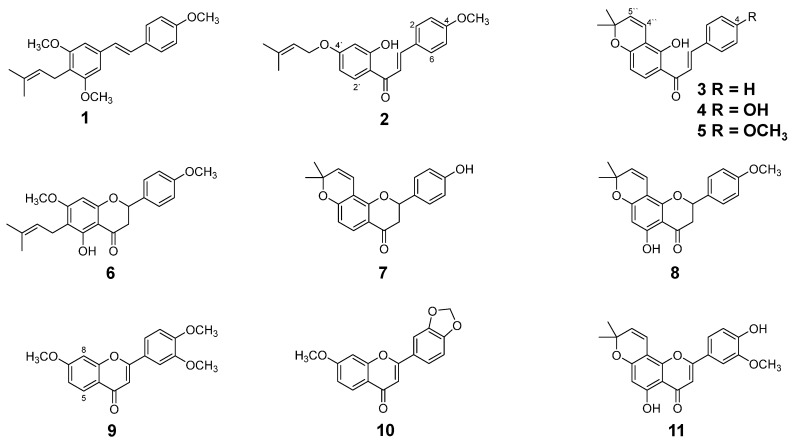
Chemical structures of isolated flavonoids from *D. duckeana*.

Fractionation of the CH_2_Cl_2_ extract from the roots of *D. duckeana* afforded eight known flavonoids. Based on ^1^H-, ^13^C-NMR and EI-MS data as well as comparison to spectral data from the literature these compounds were identified as 3,5,4’-trimethoxy-4-prenylstilbene (**1**) [11], 4-methoxy-derricidine (**2**) [11], 4-hydroxylonchocarpine (**4**) [11], 4-methoxylonchocarpine (**5**) [11], 4’-hydroxy-isolonchocarpine (**7**) [12], 4’-methoxyisolonchocarpine (**8**) [13], 3’,4’-methylenedioxy-7-methoxyflavone (**10**) [14] and 2,2-dimethylchromone-5,4’-dihydroxy-5’-methoxyflavone (**11**) [15]. The hexane extract from branches yielded two known flavonoids which were identified by the methods mentioned above as lonchocarpine (**3**) [11] and 3’,4’,7-trimethoxyflavone (**9**) [16]. Except for **1**, **3**, and **4** [11] all these compounds are described for the first time in *D. duckeana.* The structures are given in Figure 1.

Additionally, a new flavanone has been isolated as a mixture with compound **8** from the CH_2_Cl_2_ extract. EI-MS gave a molecular ion peak at *m*/*z* 368, fragment ions at *m*/*z* 353 [M − 15]^+^, 352 [M − 15 − H]^+^ and 337 [M − 31]^+^ and a base peak at *m*/*z* 203, indicating a flavonoid skeleton. ^1^H-NMR data (see Table 1) suggested the presence of a flavanone skeleton because of the signals at δ_H_ 5.34 (1H, dd, H-2) and 2.76 (1H, dd, H-3_eq_) and 3.09 (1H, dd, H-3_ax_). The presence of a γ,γ-dimethyl allyl group was obvious from signals at δ_H_ 3.25 (2H, d, H-1’’), 5.19 (1H, m, H-2’’), 1.76 (3H, brs, H-4’’) and 1.81 (3H, brs, H-5’’). The occurrence of an AA’BB’ system with signals at δ_H_ 6.95 (2H, d, H-3’ and 5’) and 7.38 (2H, d, H-2’ and 6’) revealed a *para*-substituted B-ring. The 4’-OH was methylated, because of the slight downfield-shift of H-2’/6’ and H-3’/5’ and C-4’ compared to the NMR data reported for the unmethylated flavanone from *Feronia limonia* 5-hydroxy-2-(4-hydroxyphenyl)-7-methoxy-6-(3-methylbut-2-enyl)chroman-4-one [17]. Correlation in the HSQC and HMBC spectra allowed the unambiguous assignment of the ^13^C-NMR data (see Table 1) to the respective carbon atom and confirmed the substitution at C-6 with an isoprenyl side chain and at C-7 and C-4’ with methoxy groups. The substitution of the flavanone agreed also with the correlations in the NOESY spectrum (see Figure S3 in the Supplementary Materials). Hence, compound **6** is named as 5-hydroxy-4’,7-dimethoxy-6-prenylflavanone which has not been reported to the best of our knowledge, but was synthesized as a flavone [18].

**Table 1:** ^1^H-NMR and ^13^C-NMR spectroscopic data of compound **6** (CDCl_3_, 400 MHz, *J* in Hz).

C	δ_C_ (ppm)	δ_H_ (ppm)
2	79.1	5.34 (dd, 13.0 and 3.0)
3	43.3	2.76 (dd, 17.2 and 3.0) ^a^ and 3.09 (dd, 17.2 and 13.0) ^b^
4	196.0	-
5	160.0	-
6	109.9	-
7	165.4	-
8	90.9	6.07 (1H, s)
9	161.4	-
10	102.9	-
1’	130.3	-
2’/6’	127.7	7.38 (d, 8.5)
3’/5’	114.2	6.95 (d, 8.5)
4’	160.2	-
1”	21.0	
2”	122.2	5.19 (1H, m)
3”	131.6	
4”	25.8	1.76 (3H, s)
5”	17.7	1.81 (3H, s)
5-OH	160.2	12.06 (1H, s)
7-OCH_3,_ 4’-OCH_3_	55.3	3.83 (6H, s)

^a^ H-3_eq_; ^b^ H-3_ax_.

### 2.2. The Flavonoids from D. duckeana Differently Influence Cell Viability

**Figure 2:**
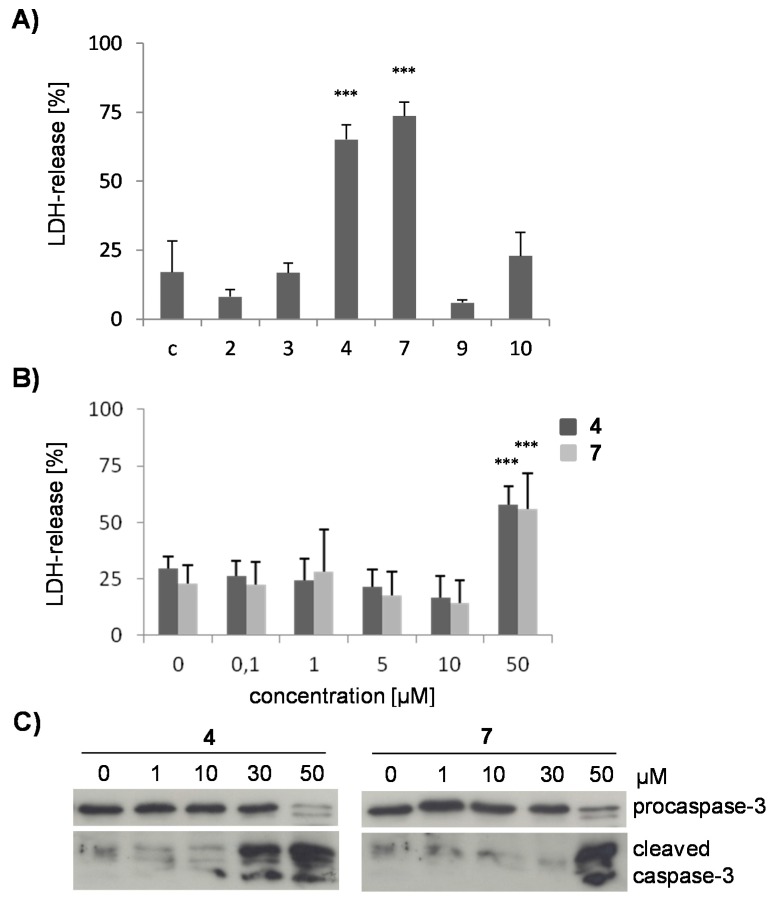
Cytotoxicity of flavonoids from *D. duckeana*. Cell damage was determined by an LDH-release assay after incubating SK-N-SH cells with the flavonoids **2**–**4**, **7**, **9**, and **10** at 50 µM (**A**) or with the compounds **4** and **7** at concentrations from 0.1–50 µM (**B**) for 24 h. Values represent means ± s.d. of three (**A**) or four (**B**) independent experiments. Statistical evaluation was performed by one-way ANOVA (in **A**) or two way ANOVA (in **B**), followed by the Bonferroni *post hoc* test. ***, *p* < 0.001 *vs.* untreated control cells. In (**C**), SK-N-SH cells were treated with 1–50 µM of compound **4** or **7** for 6 h before cellular lysates were analyzed for pro-caspase-3 cleavage by immunoblotting. A representative blot is shown (*n* = 3).

Because timbó is considered as neurotoxic to fish, the biologic effects of isolated compounds were tested in the neuronal cell line SK-N-SH. Chalcones are known for their cytotoxic activity [4], and the compounds **2**, **3** and **4** were first evaluated for their cytotoxic potential. To study whether the 2’’,2’’-dimethylpyrano- or the 3’,4’-methylenedioxy structural elements play a role in the cytotoxic activity compounds **7**, **9** and **10** were included in the study. The cells were incubated with the respective flavonoid (50 μM) for 24 h and cell death was determined by measuring the release of intracellular lactate dehydrogenase into the supernatant (Figure 2A,B). Only the chalcone **4** and the flavanone **7**, which both possess a 4’-hydroxy group and a 2’’,2’’-dimethylpyrano moiety, induced significant membrane damage, whereas the chalcones **2**, **3** and the flavones **9** and **10** mediated no cytolytic effect. Compounds **4** and **7** were studied for their LDH release at different concentrations. No significant cell death was observed up to a 10 μM concentration, but only at 50 μM (Figure 2B).

To test whether cell death induced by compounds **4** and **7** is due to apoptosis, activation of caspase-3 was determined by immunoblotting. Cleavage of pro-caspase-3 to its active fragments was observed (Figure 2C), indicating that cytotoxicity, as determined in Figure 2A,B, may be due to secondary necrosis and a consequence of apoptosis.

**Figure 3:**
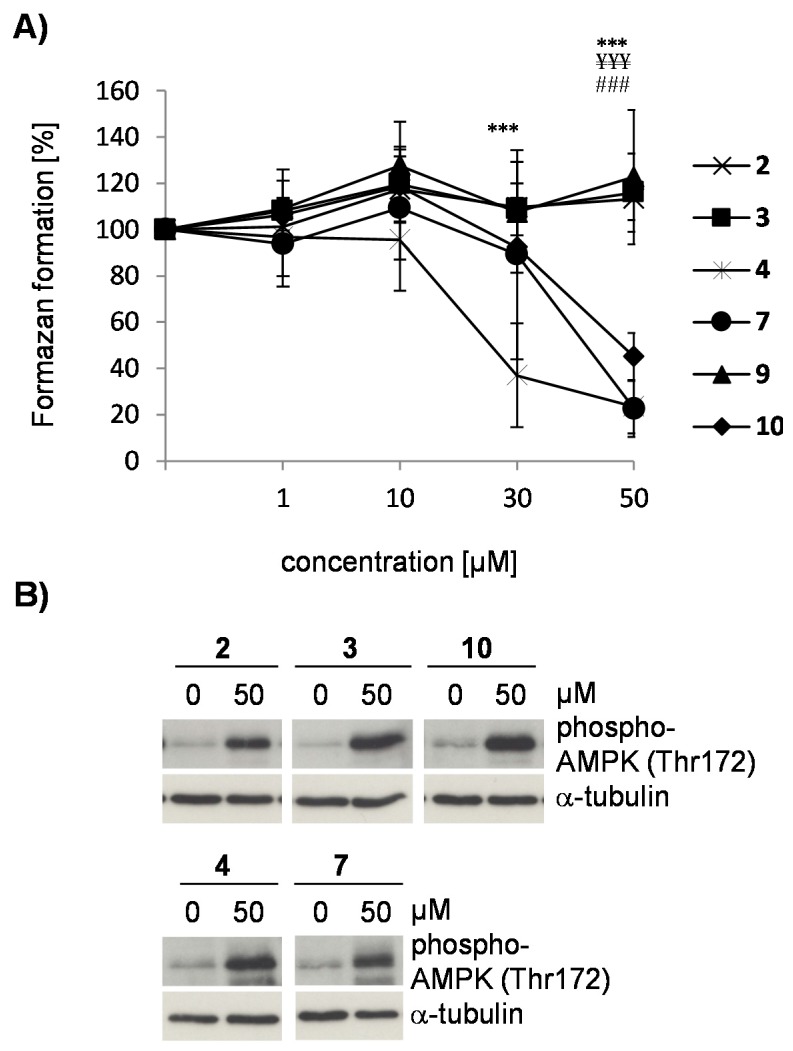
Effect of flavonoids **2**, **3**, **4**, **7**, **9**, and **10** on cellular metabolism. In (**A**), an MTT cell viability assay was performed. Cells were pretreated with 1–50 µM of the respective flavonoid for 24 h. Formazan formation was compared to DMSO-treated control cells (=100 %). Values represent means ± s.d. of five independent experiments. Statistical evaluation was performed by two-way ANOVA followed by the Bonferroni’s *post hoc* test. Statistically significant differences are shown for cells treated with compound **4** *vs.* untreated cells (***, *p* < 0.001), compound **7** *vs.* untreated cells (^¥¥¥^, *p* < 0.001) and compound **10** *vs.* untreated control (^###^, *p* < 0.001). In (**B**), SK-N-SH cells were treated with 50 µM of compound **2**, **3**, **4**, **7** or **10** for 6 h, before cell lysates were analyzed for AMPK phosphorylation at threonine 172 by immunoblotting (upper blot). Immunoblots were reprobed for α-tubulin to demonstrate the analysis of equal amounts of protein in each sample (lower blot). Representative blots are shown (*n* = 3).

Results were verified by the MTT cellular viability assay (Figure 3A). At concentrations ≥30 µM for compound **4** and 50 µM for compound **7**, treated cells displayed a significantly reduced conversion of 3-(4,5-dimethylthiazol-2-yl)-2,5-diphenyltetrazolium bromide (MTT) to its formazan salt, indicating a decline in cellular viability. These concentrations are sufficient to induce apoptosis (Figure 2C) and secondary necrosis (Figure 2A,B). In contrast, compounds **2**, **3**, and **9** did not reduce conversion of the tetrazolium salt, confirming that these compounds have no effect on cellular viability (Figure 3A). Interestingly, the flavone **10** which differed from compound **9** by the 3’,4’-methylenedioxy group diminished the reducing capacity of SK-N-SH cells, as observed by a decline in absorbance (Figure 3A) without simultaneously killing the cells (Figure 2A). Reduced formazan conversion in the absence of cell death has been associated with impaired metabolism [19]. Despite the limiting data for drawing structure activity relationships the occurrence of the 3’,4’-methylenedioxy group may be important for a potential decrease of the metabolic activity in SK-N-SH cells.

### 2.3. Flavonoids from D. duckeana Induce the Phosphorylation of AMPK and eEF2, But Not eIF2alpha

Cells with a low metabolism reduce very little MTT [20]. Therefore, we tested whether the flavone **10** attenuates the cellular metabolism by determining activation of AMPK, an energy-sensing enzyme closely involved in the regulation of energy homeostasis [21]. As shown in Figure 3B, treatment of cells with compound **10** resulted in an increase in AMPK-phosphorylation on its activating loop at threonine-172. Phosphorylation of AMPK at threonine-172 is a consequence of a conformational change due to increased AMP concentrations [22] and a prerequisite of AMPK to adjust intracellular energy levels by inhibiting energy consuming pathways such as global protein synthesis [23,24]. However, compounds **2**, **3**, **4** and **7** also induced phosphorylation of AMPK (Figure 3B) indicating that reduced formazan formation (Figure 3A) and AMPK activation are here independent processes.

**Figure 4:**
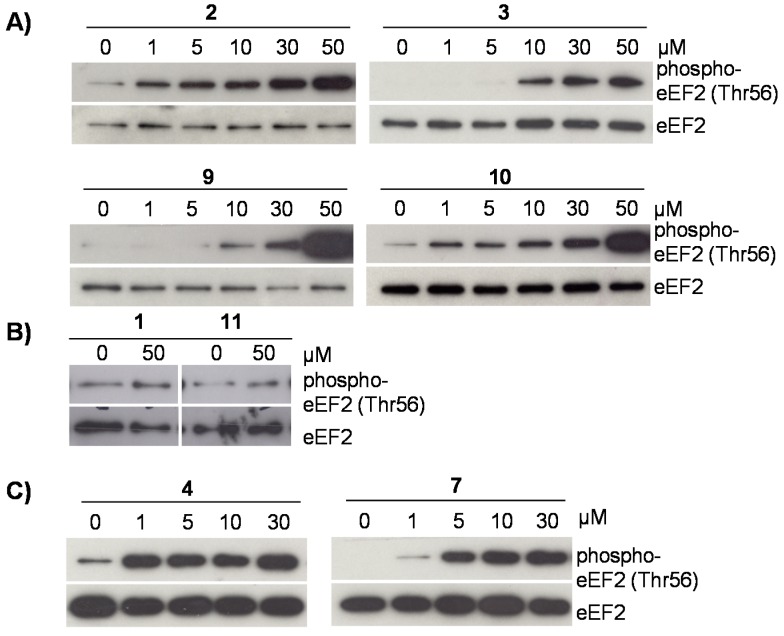
(**A**–**C**) Effect of flavonoids **1**–**4**, **7**, **9**–**11** on translational elongation. Phosphorylation of eEF2 at threonine 56 was analyzed in lysates of cells, pretreated with 1–50 µM of the indicated flavonoids for 6 h by immunoblotting. Immunoblots were reprobed for total eEF2 to visualize that similar amounts of proteins were analyzed in each sample. Representative blots are shown (*n* = 3).

Activation of AMPK indicates inhibition of global protein synthesis [23]. To study a possible impact of the tested compounds on translation, we determined their potential to induce phosphorylation of eEF2 at threonine 56, which blocks translational elongation by an AMPK/eEF2K-dependent mechanism and thus protein synthesis [23]. As shown in Figure 4A, the flavonoids **2**, **3**, **4**, **7**, and **10** markedly induced phosphorylation of eEF2, which couples AMPK activation with reduced translational elongation. The stilbene **1** and the flavone **11** did not mediate this posttranslational modification of eEF2 at threonine 56. However, up to now no structure activity relationships can be drawn.

The cytotoxic compounds **4** and **7** also induced posttranslational repression of eEF2 (Figure 4C), but at 50 µM eEF2 phosphorylation at threonine 56 declined in the presence of both compounds (data not shown). This is most probably due to the strong cytotoxic activity of these flavonoids (Figure 2A–C). Altogether, our results indicate, that the cytolytic potential of some flavonoids isolated from *D. duckeana* is not directly related to their potential to repress translational elongation.

**Figure 5:**
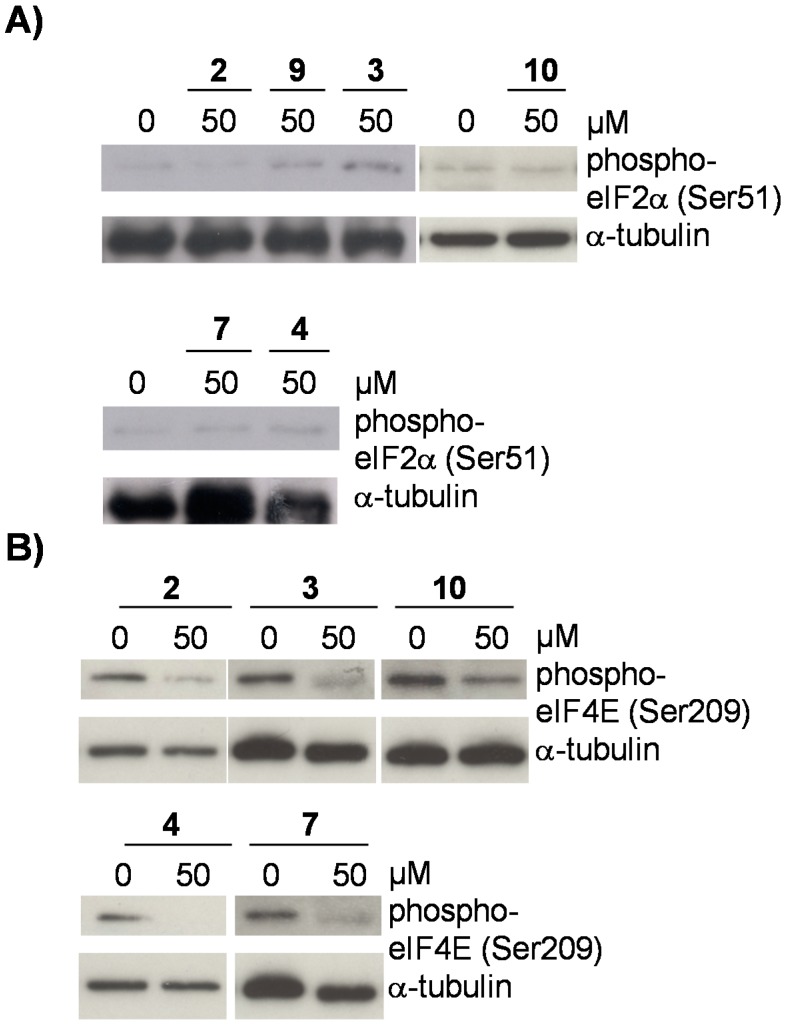
Effect of flavonoids **2**–**4**, **7**, **9**, and **10** on translational initiation. Phosphorylation of eIF2alpha at serine 51 or eIF4E at serine 209 was analyzed in lysates of cells, pretreated with 50 µM of the indicated flavonoids for 6 h by immunoblotting. Immunoblots were reprobed for α-tubulin to visualize that similar amounts of proteins were analyzed in each sample. Representative blots are shown (*n* = 3).

To test the influence of flavonoids from *D. duckeana* on the initiation phase in the translation process, phosphorylation of eIF2alpha was determined in the presence of compounds **2**, **3**, **4**, **7**, **9** and **10**. eIF2alpha is required in the initiation of translation, as it mediates binding of GTP and the initiator Met-tRNA to the ribosome to form the 43S preinitiation complex. Phosphorylation at serine 51 inactivates eIF2alpha and as a consequence translation comes to halt because initiation is abrogated. As shown in Figure 5A, none of the tested flavonoids were able to mediate posttranslational phosphorylation of eIF2alpha at serine 51. Furthermore, we tested the potential of the flavonoids **2**, **3**, **4**, **7**, and **10** on the phosphorylation of eIF4E. Activation of the eukaryotic initiation factor eIF4E is a rate limiting step in cap-dependent translation and is regulated among others by AMPK under energy starvation [25]. Our results, shown in Figure 5B, demonstrate that the flavonoids **2**, **3**, **4**, **7** and **10** repress phosphorylation of eIF4E at serine 209, which is associated with its reduced activity. Phosphorylation of eIF4E at serine 209 is mediated by the ras/MAP-kinase pathway [25], indicating that flavonoids from *D. duckeana* probably also affect mitogenic or stress stimuli via ERK1/2 or p38 MAP-kinase.

### 2.4. Conclusions

Altogether, we could extend the knowledge on the flavonoid profile of *D. duckeana* which fits those known from other species in the genus *Deguelia*. The isolated flavonoids were shown to have various effects on cells. Compounds **4** and **7**, which both possess a 2”,2”-dimethylpyrano structure, may contribute to the reported killing of fish because of their proven cytotoxic effects. Moreover, we could demonstrate for the first time that flavonoids such as **2**, **3**, **4**, **7**, **9** and **10** affect phosphorylation of eEF2, AMPK and eIF4E and thus influence both translational initiation and elongation. Resulting consequences must be clarified by further experiments to elucidate the applicability of flavonoids in prospective therapeutic settings.

## 3. Experimental Section

### 3.1. General Procedures

NMR spectra were recorded on a DRX 400 NMR instrument (Bruker, Bremen, Germany) at 400 MHz (^1^H) and on an Avance III (Bruker) at 400 MHz (^1^H) and at 100 MHz (^13^C) either in CDCl_3_ and in methanol-*d_4_*. HR-APCI-MS spectra were measured on a Thermo Q Exactive mass spectrometer with an Orbitrap-Analyzer (Thermo Scientific, West Palm Beach, FL, USA). Open column chromatography was carried out by using silica gel 60 (230–400 and 70–230 mesh, Merck, Darmstadt, Germany), Sephadex LH-20 and Florisil (100–200 mesh, Merck). The fractions were monitored by analytical TLC (Silica gel 60 F_254_, aluminium sheets, Merck) using an Automatic TLC Sampler (CAMAG, Muttenz, Switzerland).

### 3.2. Plant Material

Roots and branches from *Deguelia duckeana* A. M. G. Azevedo (Fabaceae) were collected in Praia Dourada, Manaus, Amazonas. Identification was done by the botanist Ieda Leão do Amaral. Roots were collected in April, 2006 and branches in August, 2009. Voucher specimens are deposited at the Herbarium of the Instituto Federal de Educação do Amazonas (IFAM), under the numbers 10606 and 10613, respectively.

### 3.3. Extraction and Isolation

The material from *D. duckeana* were dried for three days in a hot-air oven 50 °C and separately extracted with CH_2_Cl_2_ for roots and with *n*-hexane for branches in a 2 L flask using an ultrasonic bath (Unique, Indaiatuba, São Paulo, Brazil) for 20 minutes and filtered. The extracts were concentrated *in* *vacuo* (40 °C). The CH_2_Cl_2_ extract from the roots (8.0 g) was fractionated by open column chromatography (CC) on SiO_2_ gel 60 (230–400 mesh, 138 × 3.2 cm, 104 g of SiO_2_) and eluted with a gradient of *n*-hexane/CH_2_Cl_2_, EtOAc/MeOH and MeOH 100%. 42 fractions were obtained which were monitored by TLC (*n*-hexane/CH_2_Cl_2_ 8:2 and EtOAc/MeOH 7:3 with anisaldehyde/H_2_SO_4_ and Ce(SO_4_)_2_). Fraction 3 (200.0 mg) was separated by CC on SiO_2_ (70–230 mesh, 40 × 1.8 cm) with mixtures of CH_2_Cl_2_ and EtOAc yielding 10 fractions. Compound **1** (2.7 mg) was isolated from subfraction 6 (100.2 mg) by open CC on SiO_2_ (70–230 mesh, 26 × 1 cm, 20 g) with CHCl_3_ and a mixture of CHCl_3_/MeOH (75:5). Fractions 4 and 5 were combined (2.8 g) and were subjected to open CC on SiO_2_ (230–400 mesh, 74.5 × 3 cm) using mixtures of *n*-hexane, EtOAc and MeOH. 50 subfractions were obtained and monitored by TLC. Subfractions 13 to 15 (276.0 mg) afforded mainly compound **7**, subfractions 16 to 18 (151 mg) were enriched with compound **2**, subfraction 19 (30.0 mg) contained a mixture of compounds **2**, **5** and **11** and subfraction 27 (15.0 mg) was enriched with compound **10**. Compound **7** (9.6 mg) was purified on Sep-Pak Cartridges using Florisil (10.0 g, Phenomenex, Torrance, CA, USA) and a mixture of CH_2_Cl_2_ and MeOH, followed by open CC on SiO_2_ (230–400 mesh, 30.0 g) and *n*-hexane, CH_2_Cl_2_ and MeOH as solvent. Compound **2** (5.3 mg) was isolated from subfraction 16–18 by open CC on SiO_2_ (230–400 mesh, 40 × 1.8 cm) with mixtures of *n*-hexane, CH_2_Cl_2_ and EtOAc, followed by preparative TLC using the system CH_2_Cl_2_/EtOAc (95:5, *v*:*v*). Compound **11** (1.0 mg) was isolated from subfraction 19 by precipitation from MeOH. The remaining part of subfraction 19 was fractionated twice on Sephadex LH-20 column (2 × 10.5 cm) using MeOH followed by preparative TLC on SiO_2_ using the system CHCl_3_/MeOH (75:5, *v*:*v*). After open CC on Sephadex LH-20 (12.5 × 0.5 cm) a mixture (1.6 mg) of compounds **2** and **5** was obtained. Fraction 8 (30.2 mg) from the first separation procedure was again separated by CC on SiO_2_ (70–230 mesh, 20 × 1 cm) using mixtures of CHCl_3_/MeOH (78:2, 75:5) and MeOH 100% and afforded a fraction with a mixture of compounds **6** and **8**. Fraction 21 (2.35 g) obtained from the first open CC was further fractionated by open CC on SiO_2_ (70–230 mesh, 20 × 3.6 cm) with a gradient of CH_2_Cl_2_, EtOAc and MeOH, followed by an open CC on SiO_2_ (230–400 mesh, 150 × 1.47 cm) and a gradient of *n*-hexane/CH_2_Cl_2_ and EtOAc and subsequently by an open CC on SiO_2_ (230–400 mesh, 140 × 1.8 cm, 88.75 g) and a gradient of CH_2_Cl_2_, EtOAc and MeOH. Compound **4** (1.0 mg) was finally obtained by precipitation from MeOH.

The hexane branch extract (2.0 g) was subjected to open CC on SiO_2_ (140 × 3.1 cm) using mixtures of *n*-hexane, EtOAc and MeOH and 112 fractions were yielded. Subfraction 25 (180.0 mg) was separated by open CC on neutral alumina (40 × 1.8 cm) using mixtures of *n*-hexane, CH_2_Cl_2_ and MeOH. Subfraction 9 (7.0 mg) was fractionated by preparative TLC (SiO_2_ gel 60 F_254_ plates) using *n*-hexane/CH_2_Cl_2_ (3:7, *v*:*v*) and afforded compound **3** (4.0 mg). Subfraction 63–66 (15.0 mg) was subjected to open CC on SiO_2_ (70–230 mesh, 20 × 0.5 cm, 5 g) using mixtures of CHCl_3_/MeOH (9:1, 8:2, 7:3) yielding compound **9** (3.1 mg). Fractionation schemes are shown in the Supplementary Materials (Figures S1 and S2).

*Hydroxy-4’,7-dimethoxy-6-prenylflavanone* (**6**): EI-MS: *m*/*z* (rel. int. %) 368 [M − 15]^+^ (33), 353 [M − 15]^+^ (16), 352 [M − 15 − H]^+^ (30), 337 [M − 31]^+^ (54), 218 (16), 203 (100), 179 (23); ^1^H-NMR, ^13^C-NMR: Table 1, HMBC and NOESY (Figure S3).

### 3.4. Cell Culture and Treatment

The human neuronal cell line SK-N-SH was obtained from the American Type Culture Collection (Manassas, VA, USA) and maintained in Eagle’s minimal essential medium (EMEM), supplemented with 10% fetal bovine serum (FBS), 100 IU/mL penicillin and 100 μg/mL streptomycin at 37 °C in a humidified atmosphere containing 5% CO_2_ (Thermo Fisher Scientific, Waltham, MA, USA). Cells were treated with 0.1–50 µM of the flavonoids after synchronization in FBS-free EMEM containing 100 IU/mL penicillin and 100 µg/mL streptomycin overnight. Control cells were treated with the highest concentration of DMSO as solvent (0.1%).

### 3.5. Cytotoxicity Assay

SK-N-SH cells (1 × 10^6^ cells per well of a 6 well plate) were treated with the flavonoids for 24 h, before cellular supernatants were analyzed for lactate dehydrogenase (LDH) content by the Cytotoxicity Detection Kit (Roche Applied Science, Mannheim, Germany) according to the protocol of the manufacturer. The absorbance at 490 nm was measured by a microplate reader (Model 680, BIO-RAD, Munich, Germany) with the reference wavelength of 690 nm. Total LDH release (100%) was obtained by the treatment of cells with 2% Triton-X100. The relative LDH release is defined by the ratio of LDH released over total LDH in the intact cells.

### 3.6. Metabolic Activity

The MTT viability assay was performed as described by Mosmann [20] Briefly, SK-N-SH cells (50000 cells per well of a 96 well plate) were treated with the respective flavonoid for 24 h and subsequently incubated with 1 mg/mL of 3-(4,5-dimethylthiazol-2-yl)-2,5-diphenyl tetrazolium bromide (MTT) for 2 h at 37 °C before excess MTT was aspirated. The residual formazan crystals were dissolved in 100 μL of dimethylsulfoxide and quantified at 595 nm, using a microplate reader (Model 680, BIO-RAD, Munich, Germany).

### 3.7. Immunoblot Analysis

SK-N-SH cells (50000 cells per well of a 96 well plate) were treated with the flavonoids for 6 h and subsequently lysed in 5 × SDS electrophoresis sample buffer. Lysates were sonicated and boiled for 3 min before proteins were separated by SDS-polyacrylamide gel electrophoresis, transferred onto a polyvinylidene difluoride membrane (EMD Millipore, Billerica, MA, USA) and blocked with 5% dry milk in Tris-buffered saline. Antibodies, raised against caspase-3, cleaved caspase-3, phospho-AMPK (Thr172), phospho-eEF2 (Thr56), eEF2, phospho-eIF2alpha (Ser51), phospho-eIF4E (Ser209) or α-tubulin were used for detection of specific proteins (Cell Signaling Technology, Danvers, MA, USA). After repeated washing, the specific bands were visualized using horseradish peroxidase-conjugated anti-rabbit IgG and enhanced chemiluminescence reagents (GE Healthcare, München, Germany).

### 3.8. Statistical Analysis

Data are shown as mean ± s.d. Statistical analysis was performed using the GraphPad Prism 5 software (GraphPad, La Jolla, CA, USA) and one-way or two way analysis of variance (ANOVA) followed by the Bonferroni *post hoc* test. Results were considered significant with *p* ≤ 0.05.

## Supplementary Materials

Schemes of *Deguelia* *duckeana* fractionation are available online at http://www.mdpi.com/1420-3049/21/2/192/s1.
