# Supplementary material for: Isolation of Flavonoids from Deguelia duckeana and Their Effect on Cellular Viability, AMPK, eEF2, eIF2 and eIF4E
Source: Molecules. 2016 Feb 6;21(2):192. doi: 10.3390/molecules21020192 (PMC6274060; doi:10.3390/molecules21020192)
Supplement: Supplementary file 1 [file molecules-21-00192-s001.pdf]

Supplementary Materials: Isolation of Flavonoids from *Deguelia duckeana* and Their Effect on Cellular Viability, AMPK, eEF2, eIF2 and eIF4E

Lorena M. C. Cursino [1,2], Nerilson M. Lima [1], Renato Murillo [3], Cecilia V. Nunez [1], Irmgard Merfort [2,*] and Matjaz Humar [2,*]

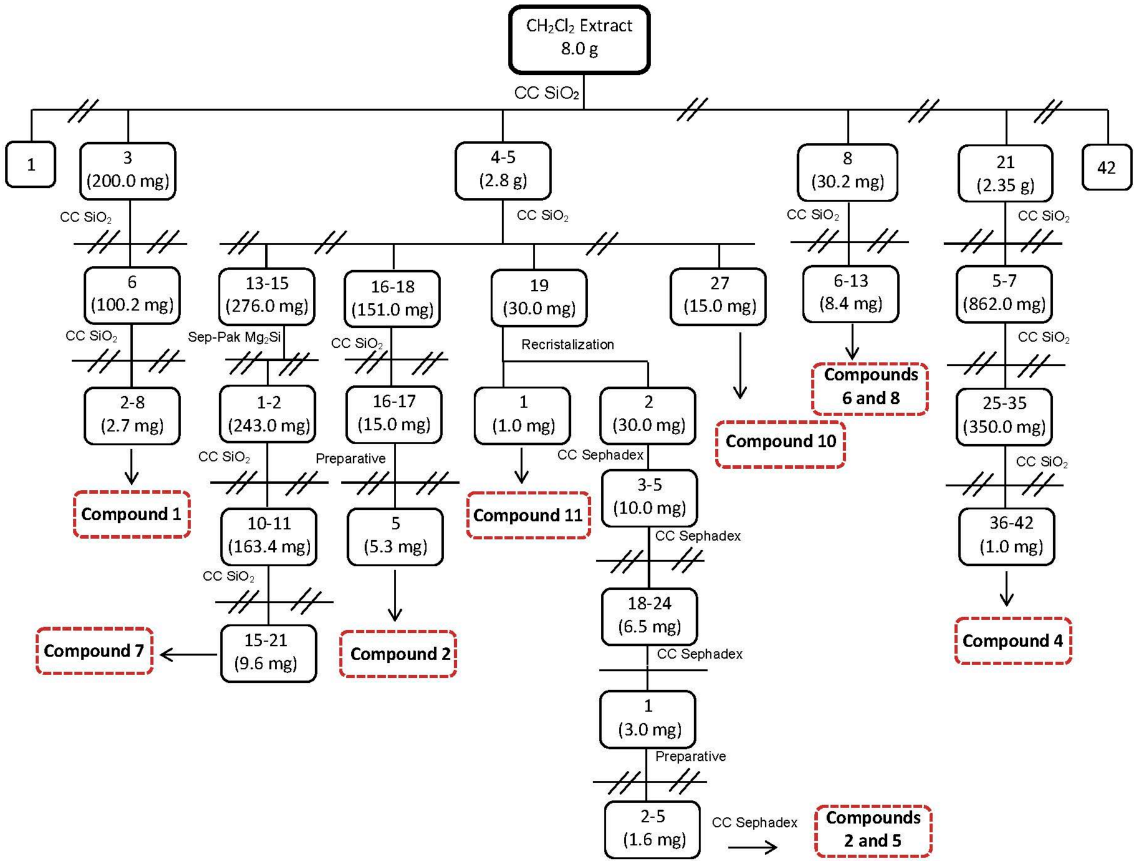

Figure S1. Chemical fractionation of dichloromethane extract of *Deguelia duckeana* roots.

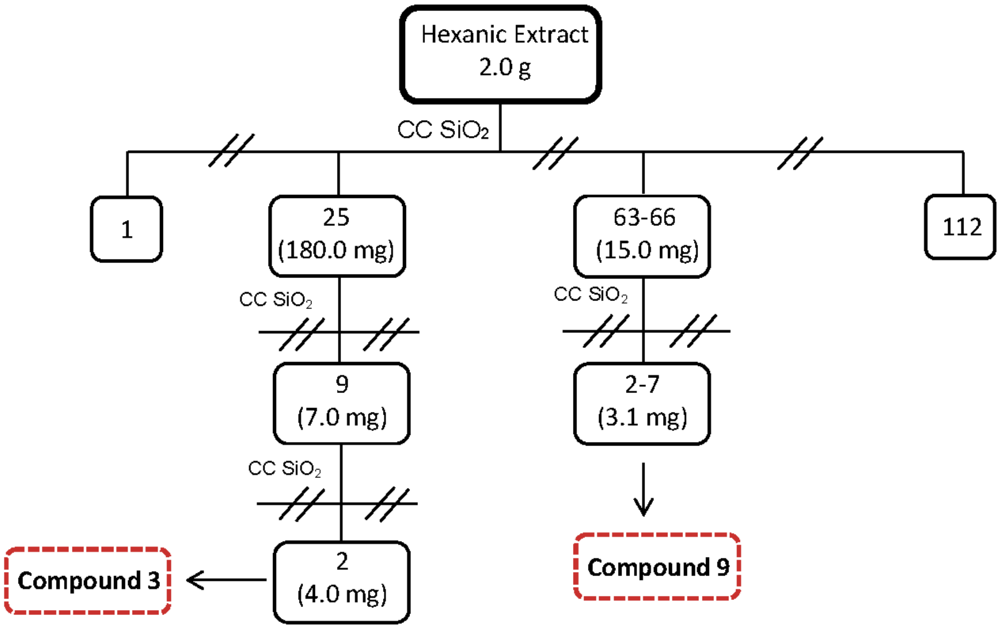

Figure S2. Chemical fractionation of hexanic extract of *Deguelia duckeana* branches.

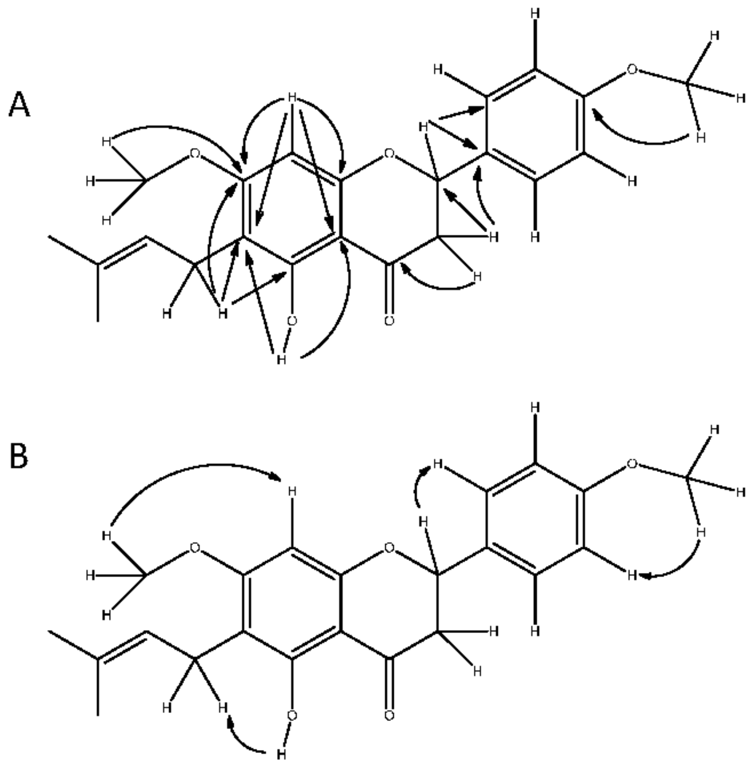

Figure S3. HMBC correlations (**A**) and NOE correlation (**B**) of compound **6**.
